# Bioluminescent *Xanthomonas hortorum* pv. *gardneri* as a Tool to Quantify Bacteria *in Planta*, Screen Germplasm, and Identify Infection Routes on Leaf Surfaces

**DOI:** 10.3389/fpls.2021.667351

**Published:** 2021-06-15

**Authors:** Eduardo Bernal, Loïc Deblais, Gireesh Rajashekara, David M. Francis

**Affiliations:** ^1^Department of Horticulture and Crop Science, Ohio Agricultural Research and Development Center, The Ohio State University, Wooster, OH, United States; ^2^Food Animal Health Research Program, Ohio Agricultural Research and Development Center, The Ohio State University, Wooster, OH, United States

**Keywords:** bacterial spot, phenotyping, tomato, *Solanum lycopersicum*, disease screening, heritability

## Abstract

Imaging technology can provide insight into biological processes governing plant-pathogen interactions. We created and used a bioluminescent strain of *Xanthomonas hortorum* pv. *gardneri* (Xg^b^) to quantify infection processes in plants using tomato as a model. An *X. hortorum* pv. *gardneri* is one of the four *Xanthomonas* species that causes bacterial spots in tomatoes. We used Xg^b^ to quantify bacterial growth *in planta*, to assess disease severity in resistant and susceptible tomato lines, and to observe infection routes in leaves. A positive and significant linear correlation r (67) = 0.57, *p* ≤ 0.0001 was observed between bioluminescence signals emitted by Xg^b^ i*n planta* and bacterial populations determined through dilution plating. Based on bioluminescence imaging, resistant and susceptible tomato lines had significantly different average radiances. In addition, there was a positive and significant correlation r = 0.45, p = 0.024 between *X. hortorum* pv. *gardneri*-inoculated tomato lines evaluated by bioluminescence imaging and tomatoes rated in the field using the Horsfall-Barrat Scale. Heritability was calculated to compare the genetic variance for disease severity using bioluminescence imaging and classical field ratings. The genetic variances were 25 and 63% for bioluminescence imaging and field ratings, respectively. The disadvantage of lower heritability attained by bioluminescence imaging may be offset by the ability to complete germplasm evaluation experiments within 30 days rather than 90–120 days in field trials. We further explored *X. hortorum* pv. *gardneri* infection routes on leaves using spray and dip inoculation techniques. Patterns of bioluminescence demonstrated that the inoculation technique affected the distribution of bacteria, an observation verified using scanning electron microscopy (SEM). We found significant non-random distributions of *X. hortorum* pv. *gardneri* on leaf surfaces with the method of inoculation affecting bacterial distribution on leaf surfaces at 4 h postinoculation (hpi). At 18 hpi, regardless of inoculation method, *X. hortorum* pv. *gardneri* localized on leaf edges near hydathodes based on bioluminescence imaging and confirmed by electron microscopy. These findings demonstrated the utility of bioluminescent *X. hortorum* pv. *gardneri* to estimate bacterial populations *in planta*, to select for resistant germplasm, and to detect likely points of infection.

## Introduction

Bioluminescence imaging has become widely used as a method to visualize and monitor molecules, cells, and protein-protein interactions *in vitro* and biological systems (Thouand and Marks, 2016). Enzyme-substrate systems emitting light are found in hundreds of luminescent organisms, including fireflies, bacteria, and marine animals (Thouand and Marks, 2016). In luminescent bacteria, the *luxCDABE* operon includes five genes, which encode enzymes that produce and oxidize substrates with blue/green light emitted as a reaction product (Fernández-Pinas and Wolk, 1994; Gupta et al., 2003; Thouand and Marks, 2016). The *luxCDABE* operon has been cloned into many phytopathogenic bacteria to monitor routes of infection, quantify bacterial populations, and assess disease resistance in different crops (Azegami et al., 2004; Vrisman et al., 2016; Du et al., 2017). Bioluminescence signals are generally well-correlated with bacterial populations *in planta* and therefore serve as a non-invasive technique to localize and measure the growth of pathogens in different pathosystems.

Image-based phenotyping is an expanding area of research with the goal of reducing time, costs, and resources used to screen and develop resistant plant varieties (Mutka and Bart, 2015; Furbank et al., 2019). Because bioluminescence imaging can measure quantitative variation in bacterial growth *in planta*, imaging systems have been leveraged to distinguish between resistant and susceptible germplasm in different crops. For example, a bioluminescent *Xanthomonas campestris* pv. *campestris* (Xcc) strain was developed to assess resistance and susceptibility in cabbage (Dane, 1993). The susceptible genotype displayed bacteria colonizing at hydathodes, whereas bacterial entry in the resistant cultivar was mainly through wounds on leaves (Dane, 1993). A bioluminescent *Xanthomonas campestris* pv. *vesicatoria* (now *X. euvesicatoria*) strain showed significant differences in growth on resistant and susceptible tomato lines (Dane and Dane, 1994), further suggesting that bioluminescence could be used to study plant-host interactions. More recently, a bioluminescent *Ralstonia solanacearum* strain causing bacterial wilt was developed to study infection in pepper lines (Du et al., 2017). The resistant pepper line restricted the multiplication of *R. solanacearum* in the roots relative to a susceptible line suggesting that visualizing bioluminescence can elucidate mechanistic differences in infection processes. These studies emphasize the potential for bioluminescence imaging to supplement field-based phenotyping approaches to identify and select germplasm.

In tomato, bacterial species can infect through natural openings or wounds. For example, *Pseudomonas syringae* causing bacterial speck on tomato colonizes and infects through stomata (Preston, 2000). Similarly, *Xanthomonas perforans* and *X. euvesicatoria*, two of the four *Xanthomonas* species causing bacterial spots in tomatoes also appear to colonize stomata (Zhang et al., 2009; Wang et al., 2017). Hydathode pores found on the margins of leaflets are another natural opening that may also be a point of entry by *Xanthomonas* species (Cerutti et al., 2019). Hydathodes are referred to as water stomata due to their function in discharging water from the inner leaf, in a process known as guttation (Chhabra et al., 1977). Guttation droplets serve as infection courts for the bacterial canker pathogen, *Clavibacter michiganesis* subsp. *michiganesis* (Carlton et al., 1998). Entry through guttation droplets on tomato has also been seen with *Salmonella enterica* Typhimurium, among other routes of entry (Gu et al., 2013). In both cases, bacteria colonized and reproduced within intercellular spaces in the leaf and moved into the vascular system. Infection of crucifers through hydathodes by *Xcc* has been well-characterized (Dane, 1993; Cerutti et al., 2017). A role for hydathode infection for *Xanthomonas* species causing bacterial spots in tomatoes remains to be described.

With bioluminescence imaging technology providing promise for quantifying pathogen populations and assessing colonization, the goal of this study was to describe the infection process by *X. hortorum* pv. *gardneri* (formerly *X. gardneri*) on tomato. We developed and utilized a *lux* operon-expressing virulent *X. hortorum* pv. *gardneri* strain to quantify bacterial populations, and observe infection processes on leaf surfaces. We confirmed and validated observations facilitated by bioluminescence using scanning electron microscopy (SEM). The main objectives of this study were (i) to quantify bioluminescence signals as a function of bacterial growth in tomato seedlings, (ii) to determine whether bioluminescence can be used to differentiate between tomato lines resistant and susceptible to *X. hortorum* pv. *gardneri*, (iii) to compare the accuracy of field disease ratings and bioluminescence seedling screening assays, and (iv) to observe *X. hortorum* pv. *gardneri* colonization of the tomato leaf surface.

## Materials and Methods

### Plant Material and Greenhouse Conditions

The germplasm used in this study was previously developed for resistance against *X. hortorum* pv. *gardneri, X. perforans*, and *X. euvesicatoria* (Bernal et al., 2020). Near Isogenic Lines (NILs), Quantitative Trait Locus 11A (QTL-11A), QTL-11A (+) *Xv3*, QTL-11B, and QTL-11C, have distinct QTL on chromosome 11, a major resistance locus *Xv3*, or a combination of both (Bernal et al., 2020). Wild tomato lines PI 114490 (*Solanum lycopersicum* var. *cerasiforme*), LA2533 (*Solanum pimpinellifolium*), NIL parent sources 01-BR-7087, FG12-433E-43, and OH087633, advanced QTL11 NILs, and susceptible control Ohio 88119 (OH88119) were used in germplasm screening experiments. The pedigree information is described in detail in Bernal et al. (2020). OH88119 was used to study bacterial colonization of leaf tissue and is the recurrent parent for the NILs.

Tomato seedlings were grown in a Biosafety Level 2 plant (BSL2P) certified greenhouse in the Department of Horticulture and Crop Science at The Ohio State University, Wooster, OH. Seedlings were grown in all experiments using standard cultural practices (Yang et al., 2005). The greenhouse temperature was maintained between 23 and 28°C under a 14-h photoperiod. Seedlings were kept in a 3 ft^3^ PVC pipe chamber covered in transparent plastic to maintain high relative humidity conducive to bacterial spots. The relative humidity inside the chamber was maintained between 70 and 95% as measured by a digital wireless thermo-hygrometer (Ambient Weather, Chandler, AZ, USA).

### Construction of Bioluminescent*X. hortorum* pv. *gardneri* (Xg^b^)

The bioluminescent kanamycin-resistant Xg^b^ strain was created using the pUWGR4 plasmid as previously described (Rajashekara et al., 2005; Deblais et al., 2018). Briefly, electro-competent cells were prepared by growing *X. hortorum* pv. *gardneri* SM761 in nutrient broth yeast extract (NBY) at 28°C for 24 h, followed by centrifugation and washing twice in sterile distilled water then once in 10% glycerol. The pellet was resuspended in 1/50th of the original volume in 10% sterile glycerol. A 100 μl aliquot of competent cells was electroporated in a 2 mm ice-cold electroporation cuvette containing 2 μl of EZ::TN/lux-kan cassette using MicroPulser (Biorad, Hercules, CA) at 2,400 V, 25 μF, and 400 Ω. EZ::TN/lux-kan cassette was prepared using the plasmid pUWGR4, as described previously (Rajashekara et al., 2005). Briefly, the pUWGR4 plasmid was digested with *PvuII*, and the 8 kb linear DNA fragment containing the *lux* operon and *kan*^*R*^ genes flanked by transposon mosaic ends was gel-purified using a QIAquick gel extraction kit. The transposome complex was prepared by mixing 200 ng of purified DNA (2 μl), 4 μl of *EZ::TN* transposase (Epicentre, Madison, WI), and 2 μ*l* of 100% glycerol, and the reaction mixture was incubated for 40 min at room temperature. Following electroporation, 900 μl Super Optimal broth with Catabolites repression (S.O.C.) medium (Hanahan, 1983) was immediately added to the electroporated cells, which were incubated at 28°C for 3 h (180 rpm). Following incubation, the cells were plated on Yeast Dextrose Carbonate (YDC) agar supplemented with 50 μg/ml kanamycin and incubated for 72 h at 28°C. Kanamycin-resistant bioluminescent colonies were further confirmed by Polymerase chain reaction (PCR) using primers specific to the kanamycin resistance gene, 1221 bp, (KAN-F: GTGAGAATGGCAAAAGCTTATGCATT and KAN-R: GAAAACAGCATTCGAGGTATTAGAAG).

### Bacterial Strain and Inoculum Preparation

Bioluminescent SM775-12 (Xg^b^) was grown in NBY medium supplemented with 50 μg/ml of kanamycin. Cultures were maintained at 28°C or stored frozen in 15% glycerol at −80°C. For spray inoculation experiments, bacteria were resuspended in autoclaved ddH_2_O and subsequently standardized to an absorbance of 600 nm = 0.15 in a 10-mm path length spectrophotometer, corresponding to a concentration of ~3.0 × 10^8^ CFU/ml. Xg^b^ suspension was sprayed to runoff with a compressed air sprayer (Preval sprayer, Coal City, IL) as described previously (Liabeuf et al., 2018). For dip inoculation, the suspension was standardized to the same concentration with the addition of 0.05% (vol/vol) Silwett L77 (PhytoTech Labs, Lenexa, KS, USA). Subsequently, tomato seedlings were inverted and submerged in the Xg^b^ suspension for 30 s in dip inoculation experiments (Gu et al., 2011).

### Bacterial Spot Evaluation in the Field

Field trials were conducted in the summer of 2016, 2017, and 2018. *X. hortorum* pv. *gardneri* strain SM775-12, the non-bioluminescent precursor isolate, was grown on nutrient yeast broth (NYB) agar media at 28°C for 48–72 h and subsequently resuspended in autoclaved ddH_2_0 with concentrations adjusted as above. The bacterial suspension was sprayed to run-off with a compressed air sprayer (Preval sprayer, Coal City, IL) in the greenhouse 1 week before transplanting to the field. Disease ratings were conducted on a per plot basis. The severity of bacterial spots was measured using the Horsfall-Barratt scale (Horsfall and Barratt, 1945) where 1 = no disease present to 12 = complete defoliation. Susceptible and resistant controls were used to monitor the progression of the disease over the growing season. Two ratings for each field conducted at time points corresponding to 80% of the fruit in plots were at the mature green (early) or ripe-fruit (late) stage of maturity (Bernal et al., 2020). The mature stage field rating was used in comparative analyses.

### Quantification of Bacterial Growth *in planta*

Tomato genotypes QTL11A (+) *Xv3* and OH88119 were selected as the resistant and susceptible lines for bacterial growth studies. The 4-week-old tomato seedlings were placed inside the humidity chamber in the greenhouse 2 days before inoculation. Seedlings were spray inoculated with the bioluminescent Xg^b^ in the morning and removed from the plastic chamber 48 h postinoculation (hpi). In the first experiment at 3, 4, 5, 8, and 9 days postinoculation (hpi), three leaflets from the second true leaf of each genotype were randomly selected, excised, and processed as described below. In the second experiment 3, 4, 5, 6, 7, 8, and 9 hpi were used as timepoints. In addition, a subset of OH88119 seedlings was spray-inoculated with the untransformed strain to compare virulence to Xg^b^. Individual leaflets were analyzed using the *in vivo* imaging system Lumina III IVIS (PerkinElmer, Inc., Waltham, MA, USA) to measure Total Flux in photons per second (p/sec) or Radiance in photons per second that leave a square centimeter of tissue and radiate into 1 unit of solid angle for a sphere, steradian (sr), (p/sec/cm^2^/sr). Each sample was measured using a 570 nm filter, and the exposure time was set at 3 min. Subsequently, each leaflet was weighed and macerated in 1 ml of ddH_2_0. Individual extracts were serial diluted, through a 10-fold dilution series, and plated in NBY media with kanamycin. As noted above, the experiment was conducted twice. Bacterial colonies were counted 26 h after plating and adjusted to log colony-forming units per gram of leaf tissue (CFU/g).

### Analysis of Tomato Germplasm With IVIS

Greenhouse germplasm screens were conducted as completely randomized experiments with three to four replications over four independent experiments. Selected NILs, wild species, and susceptible controls were randomly sown in a seedling tray and placed in the humidity chamber 4 weeks later. Seedlings were spray-inoculated with a suspension of Xg^b^, and bottom watered throughout the whole experiment. Seedlings were removed from the chamber at 48 hpi and kept on the bench in the greenhouse. Seedlings were removed from the greenhouse at 9 hpi and analyzed using IVIS. Each seedling was imaged using a 570 nm filter, and the exposure time was set at 3 min.

### Scanning Electron Microscopy Imaging of Leaf Natural Openings

To investigate the distribution of bacteria on leaflets relative to surfaces and natural openings, samples from the abaxial, adaxial, and edges of leaflets were processed and analyzed using SEM. Four seedlings of OH88119 each were either spray or dip inoculated with a suspension of *X. hortorum* pv. *gardneri* Xg^b^ as described above inside the humidity chamber. Samples were removed and analyzed at 4 hpi and 18 hpi with IVIS using 570 nm and 3-min exposure time. Subsequently, samples were prepared for SEM. All samples were processed at the Molecular and Cellular Imaging Center (MCIC) in Wooster, OH.

Infected leaf tissue sections (~1 × 1 mm) were cut from leaflets from the second true leaf. Uninfected tissue was also processed for SEM to quantify differences between natural openings on different surfaces of the leaflets. Leaf tissue samples were fixed in 3% glutaraldehyde, 2% paraformaldehyde in 0.1 M potassium phosphate buffer (PB), pH 7.0. Samples were vacuum infiltrated in fixative and placed on a tissue rocker overnight. Subsequently, samples were washed with 0.1 M PB for 15 min, then rinsed with sterile distilled water for 15 min three times. Fixed samples were dehydrated in a series of ethanol (ETOH) concentrations of 50, 70, and 90% for 15 min each. Fixed samples were then dehydrated in 100% ETOH four times for 15 min each and dried using liquid carbon dioxide. Samples were mounted on SEM stubs and coated in 15–40 mm of platinum. Samples were examined using a Hitachi Schottky field emission SU5000 microscope housed at the MCIC, The Ohio State University, Wooster, OH.

When processing images for data analysis, images were collected using the same parameters and adjusted using the Fiji Image J Software (Schindelin et al., 2012). The set scale function under “analyze” in Fiji was used to standardize any images during data collection. A stereological approach was used to quantify the number and size of natural openings, and the density of bacteria within a superimposed grid. This method was conducted using the plugin grid in Fiji (Rønn et al., 2000). Bacterial cells and natural openings on leaflets were counted using the Cell Counter plugin (Kurt De Vos, University of Sheffield; kurt.devos@iop.kcl.ac.uk).

### Bacterial Growth Analysis

The data for bacterial growth quantification based on log CFU/g and luminescent signals measured through IVIS were combined from both experiments. Data were analyzed using the fixed-effect linear model for ANOVA: *Y*_*ijk*_ = *u* + *G*_*i*_ +*T*_*ij*_ +*D*_*ik*_+ *B*_*k*_ + *e*_*ijk*_, with Y_ijk_ being the Total Flux or log (CFU/g) for the i^th^ leaflet within Genotype (G_i_) and Experiment (T_ij_) for k^th^ replicate within Experiment (B_k_) and Day (D_ik_) for the i^th^ individual within the experiment. A regression analysis was accomplished using the “lm” function in the R Core Package (R Core Team, 2017).

### Plant Germplasm Screening

The data for tomato near-isogenic lines were analyzed independently using the fixed-effect linear model for ANOVA: *Y*_*ik*_ = *u* + *G*_*i*_ + *B*_*k*_ + *e*_*ik*_, with y_ik_ the radiance measured by IVIS the i^th^ individual in the k^th^ block (B_k_). In a different model, we estimated the best linear unbiased predictors (BLUPs) for radiance measured by IVIS but included multiple plant genotypes, including NILs, parents, and wild sources for all experiments. BLUPs were estimated to normalize the data across the four experiments because the design was unbalanced; all genotypes were not replicated in all experiments. BLUPs were calculated using the random model: *Y*_*ijk*_ = *u* + *G*_*i*_ +*E*_*j*_ + *B*_*k*_ + *e*_*ijk*_, with Y_ijk_ the value of the phenotypic trait, for the i^th^ individual within a genotype (G_i_) for the j^th^ experiment (E) and B_k_ for replicate within the experiment (B_k_). The variance explained by genotype for radiance measured by IVIS was calculated based on the random model. We also estimated BLUPs for disease ratings for the same plant genotypes evaluated in *X. hortorum* pv. *gardneri*-inoculated field trials were conducted between 2016 and 2018. The random model was: *Y*_*ijk*_ = *u* + *L*_*i*_ +*Y*_*j*_ +*R*_*k*_ + *e*_*ijk*_, with Y_ijk_ being the field rating, for the i^th^ individual within a genotype (L_i_) for the j^th^ field year (Y_j_) and k^th^ for replicate within a year (R_k_). BLUPs were estimated using the function “ranef” in the lme4 package version 1.1.18.1 (Bates et al., 2015).

Heritability estimates for both IVIS and field ratings were calculated using the formula $H=\;\frac{\sigma_{G}^{2}}{\sigma_{G}^{2}+\;\frac{\sigma_{E}^{2}}{n}}$ where $\sigma_{G}^{2}$ and $\sigma_{E}^{2}$ are the estimates of variance for the genotype and residual error, n is the number of experimental or year replications (Cotteril, 1987). Main effects for experiments or years were dropped as described by Cotteril (1987). In addition, we calculated Reliability for radiance and field disease ratings using the formula $i^{2}=\;\frac{\sigma_{G}^{2}}{\sigma_{G}^{2}+\sigma_{Y}^{2}+\sigma_{E}^{2}}$, where $\sigma_{Y}^{2}$ is the variance for either year or experiment (Bernardo, 2020).

### Analysis of Natural Openings and Bacterial Colonization

To analyze the spatial distribution of natural openings and bacteria, we used stereological principles and tested the null hypothesis of spatial randomness using a chi-square distribution (Rønn et al., 2000; Mazzucato et al., 2008). The stereological analysis is used to obtain quantitative data from three-dimensional images by superimposing two-dimensional grids. A 140 μM^2^ grid with four quadrants was used to quantify the size and number of natural openings on the abaxial, adaxial, and tip of leaf lobes. The sampling was done using the same four quadrants in every biological replicate. To quantify the number of bacteria the same approach was applied, with a 30 μM^2^ grid having nine quadrants. The grid size was reduced to accommodate the size difference between bacteria and natural openings and the need to increase the magnification of images to visualize bacteria.

Data for the size of natural openings on the abaxial and edge of leaflets were analyzed using the fixed-effect linear model for ANOVA: *Y*_*ik*_ = *u* + *L*_*i*_ + *B*_*k*_ + *e*_*ik*_, with y_ik_ being the length or width (uM) for the i^th^ natural opening within the location of the leaflet (L_i_) for k^th^ replicate within the experiment (B_k_). Data were analyzed using the “lm” function in the R core package version 3.5.1 (R Core Team, 2017).

A chi-square analysis (χ^2^) was used for specific comparisons including the type of inoculation method, time-points, and distribution of bacteria on the abaxial, adaxial, or leaf margin. First, we utilized a χ^2^ analysis to determine if there were statistically significant differences in bacterial densities depending on the type of inoculation method (df, 1). Second, we tested the null hypothesis that *X. hortorum* pv. *gardneri* populations are randomly distributed on leaf surfaces. With different inoculation methods and time points, we conducted four chi-square analyses (df, 2) to test the random distribution of bacteria on different leaf surfaces. All χ^2^ analyses were conducted using the function “chisq.test” in the R core package (R Core Team, 2017).

## Results

### Verification of Bioluminescent *X. hortorum* pv. *gardneri* Transformation

**Figure 1:**
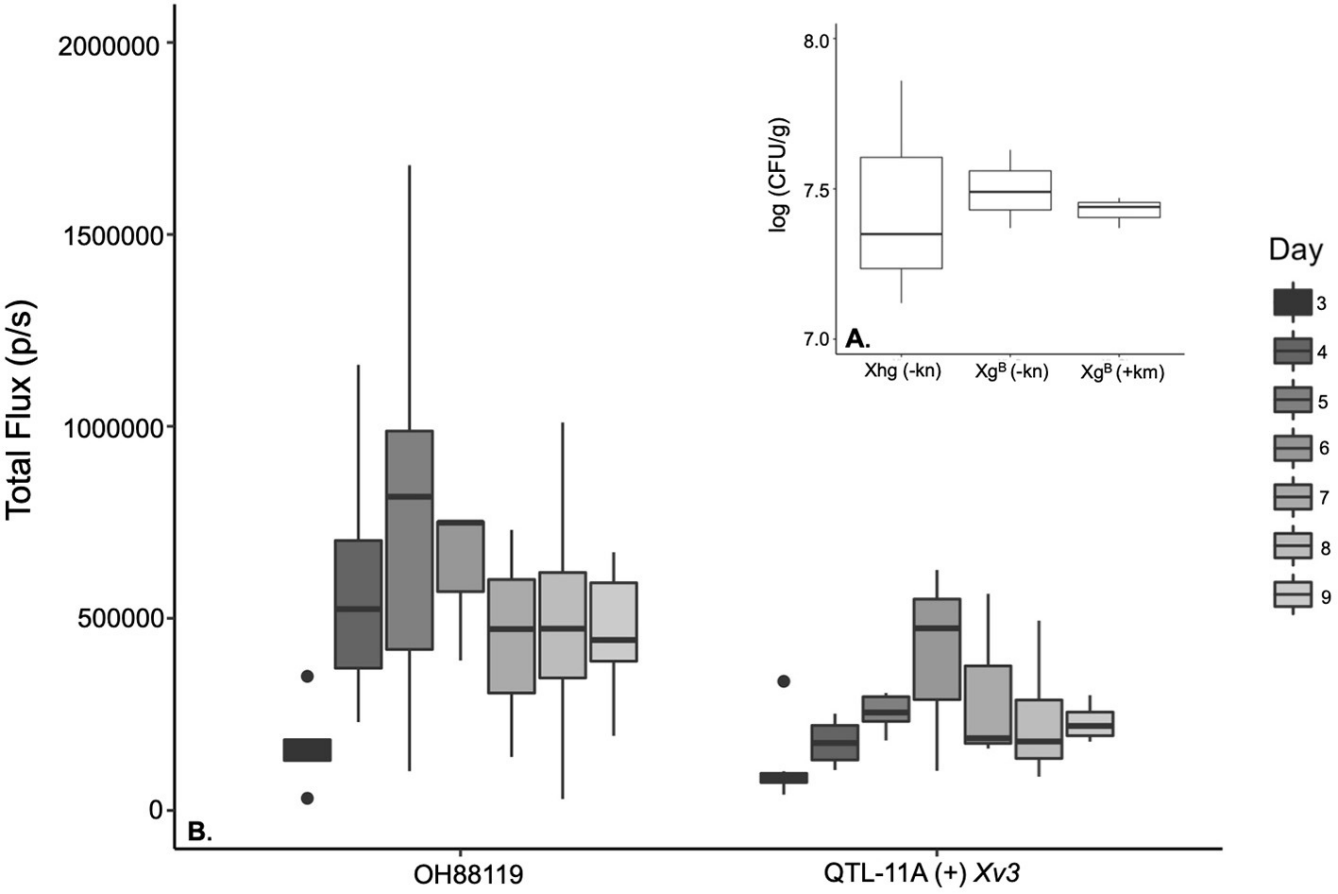
Boxplots comparing the growth of *Xanthomonas hortorum* pv. *gardneri* Xg^b^ and wild-type strain (Xhg) measured as log colony-forming units per gram of leaf tissue (CFU/g) in susceptible tomato and illustrating the population changes over time as Total Flux measured through *in vivo* imaging system (IVIS) for susceptible and resistant near-isogenic tomato lines. **(A)** No significant differences were observed for *X. hortorum* pv. *gardneri*. Xhg compared to the *lux* operon transformed strain Xg^b^ based on dilution plating and log CFU/g counts [*F*_(2, 4)_ = 0.059, *p* = ≤ 0.94]. Bacterial colonies were counted on nutrient yeast broth (NYB) media with (+) and without kanamycin (–). **(B)** illustrates the Total Flux due to Xg^b^ growing on the susceptible genotype OH88119 and the resistant Near Isogenic Lines (NIL), QTL-11A + *Xv3* through a time course.

Bioluminescent transformants were confirmed using an IVIS Model 100 (PerkinElmer, Waltham, MA, USA) and by PCR amplification targeting the kanamycin resistance gene (837 base pairs). *In vitro* insertion stability of the transposable cassette was confirmed by serial passaging (cycles of 24 h, repeated 10 times; approximately 50 generations) at 28°C without antibiotic, as previously described by Deblais et al. (2018). Bacterial growth on LB and MMX broth was not different when Xg^b^ and Xgh (wild-type SM775-12) were compared (Srivastava et al., 2021). There were no differences observed between Xgh and Xg^b^ bacterial populations in OH88119 based on dilution plating on NYB media with and without kanamycin and log CFU/g counts (Figure 1A).

### Quantification of *X. hortorum* pv. *gardneri* Populations by IVIS and Dilution Plating

A higher level of bioluminescent signals was observed in the susceptible line OH88119 compared to resistant line QTL-11A (+) *Xv3*. Total Flux (p/s) was highest on day 5 and day 6 for OH88119 and QTL-11A (+) *Xv3* (Figure 1B). A positive and significant linear correlation was observed between bioluminescent signals (Total Flux) from inoculated tomato seedlings and bacterial population determined by dilution plating. There were significant differences for genotype: the resistant line QTL-11A (+) *Xv3* displayed lower Total Flux levels relative to the susceptible line OH88119 *F* (1,55) = 16.60, *p* = ≤ 0.001. Significant differences for genotype were also found when assessing CFU/g of leaf tissue *F*_(1, 55)_ = 10.12, *p* = ≤ 0.002. There were no significant differences between experiments using total flux measured by IVIS to assess bacterial growth *in planta F*_(1, 55)_ = 0.15, *p* ≤ 0.73. However, we did see significant differences in colony counts in different experiments.

**Figure 2:**
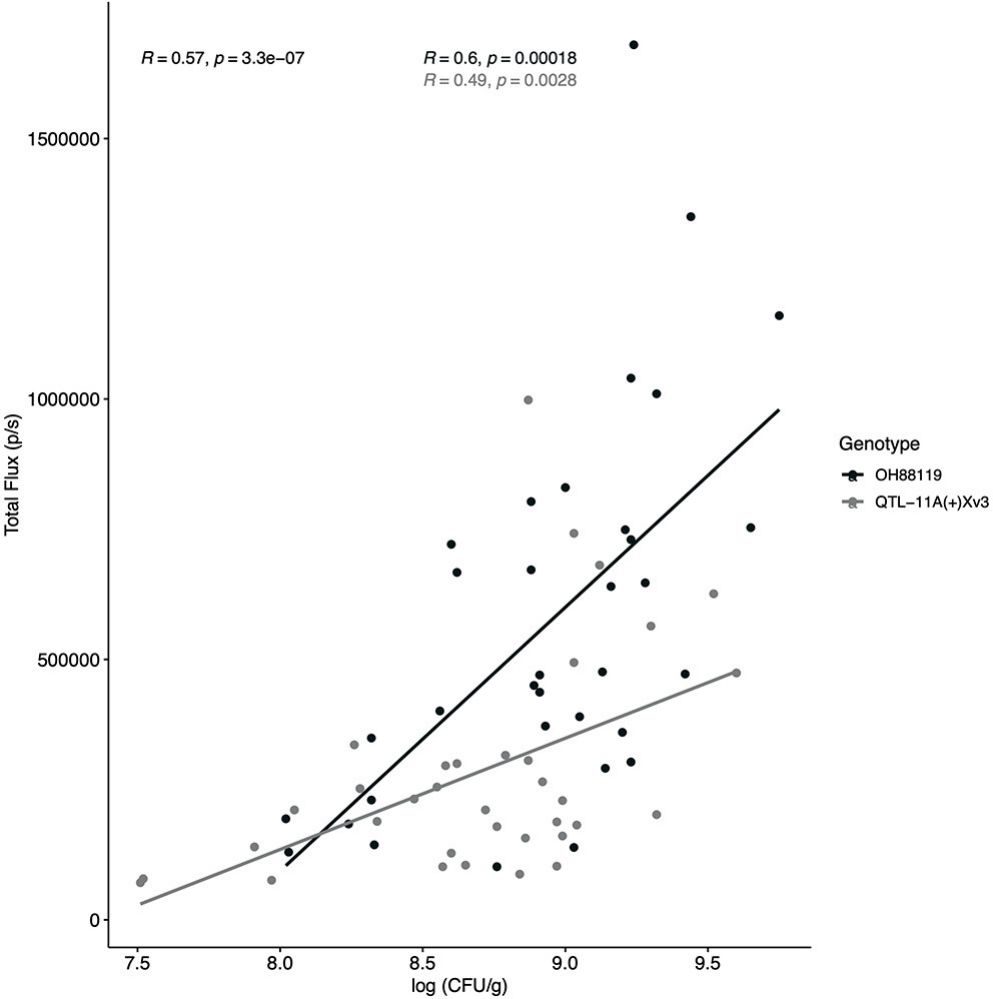
Regression analysis between Total Flux (p/s) measured through IVIS and bacterial populations. An *X. hortorum* pv. *gardneri* strain, Xg^b^, expressing the *lux operon* was developed to visualize and quantify bacteria *in planta*. Regression analysis was conducted to compare total flux (p/s) as measured through IVIS and log_10_ of bacterial populations in tomato leaf tissue (CFU/g). Bacterial colonies were counted at seven time points using resistant NIL, QTL-11A + *Xv3*, and the susceptible genotype OH88119. The regression experiment was conducted twice, the figure represents all data points. A significant and positive relationship was observed for combined data (R = 0.57, *p* < −0.0001) resistant NIL (gray line and points; R = 0.49, *p* < −0.0028) and susceptible, OH88119 (black line and points; R = 0.6, *p* < −0.0002).

A regression analysis was conducted across all time points between bacterial colony-forming units/ml and Total Flux. A positive and significant correlation r (67) = 0.57, *p* ≤ 0.0001 was observed between Total Flux (p/s) and log colony forming unit per gram (CFU/g) when all resistant and susceptible data points (*n* = 69) were combined (Figure 2). Three data points were not included because no bacterial colonies were present after plating. Independently resistant and susceptible genotypes also displayed a positive and significant correlation between log CFU/g and IVIS r (33) = 0.49, *p* ≤ 0.0003, and r (32) = 0.60, *p* ≤ 0.0002, respectively (Figure 2). Differences in bacterial growth measured by log CFU/g were seen between resistant and susceptible genotypes in the young seedlings with resistant QTL11A + *Xv3* having a lower slope relative to susceptible OH88119.

### *In vivo* Imaging System vs. Field Screening of Genotypes

**Figure 3:**
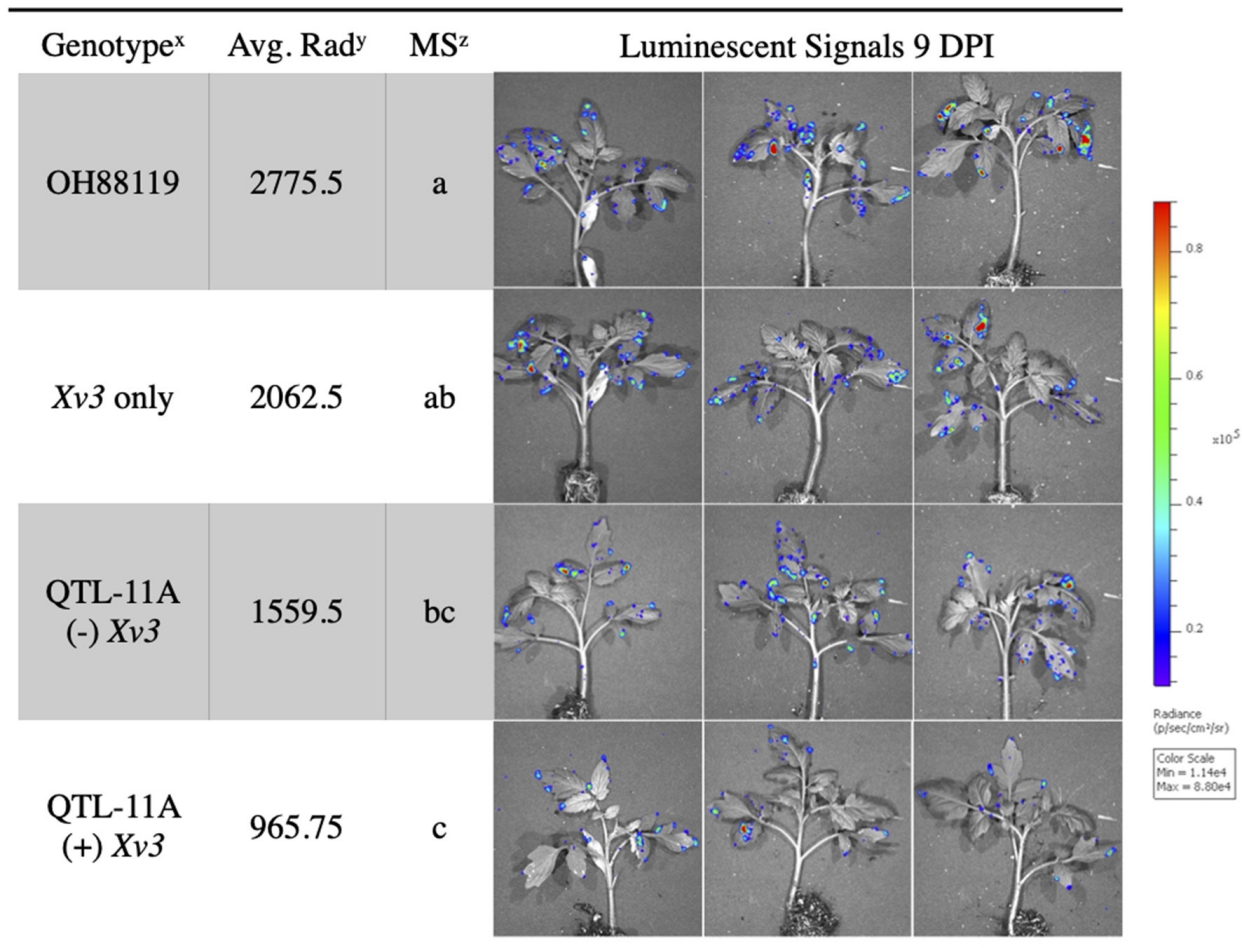
Average radiance measured through IVIS of resistant NILs and susceptible control OH88119 spray inoculated with *lux operon* expressing *X. hortorum* pv. *gardneri* (Xg^b^). Seedlings were spray inoculated and luminescent signals were measured 9 days post-inoculation. The luminescence quantification scale is located on the right. Genotype^x^ = QTL-11A (+) *Xv3* contains both a QTL and gene of resistance, QTL-11A (–) *Xv3*, contains only QTL-11A, *Xv3* only has no QTL, OH88119 is the susceptible control. Average Rad^y^ = is the average radiance (p/sec/cm^2^/sr) for four replicates of genotype. Mean Separation^z^ = represents the Fisher's Least Significance test (α = 0.05) performed following ANOVA (protected LSD).

*In vivo* imaging system measurements of average radiance differentiated between resistant NILs and susceptible lines. The fixed effect ANOVA model comparing NILs detected significant differences for genotype *F*_(3, 9)_ = 10.14, *p* = ≤ 0.003. The replicate effect was insignificant *F*_(3, 9)_ = 0.46, *p* = ≤ 0.71. Fisher's LSD was conducted to show differences between specific NILs. Tomato genotype OH88119, the susceptible control, displayed significantly higher average radiance than genotypes QTL11A only and QTL11A (+) *Xv3* inoculated with *X. hortorum* pv. *gardneri* Xg^b^ (Figure 3). We observed bioluminescent signals on the edge of the leaf suggesting that bacteria may be colonizing through hydathodes on the leaf margin (Figure 3).

Best linear unbiased predictors for IVIS average radiance and *X. hortorum* pv. *gardneri* bacterial spot field ratings were estimated for NILs, parent sources, and wild species in combined experiments. In both phenotypic approaches, OH88119 displayed the most disease severity. In contrast, wild species PI 114490 showed a high level of resistance. The comparison of BLUPs for *X. hortorum* pv. *gardneri* bacterial spot field ratings with IVIS average radiance showed a positive and significant correlation r (9) = 0.45, *p* ≤ 0.024 between data collected from both systems (Table 1).

**Table 1:** Comparison of BLUPs estimated using IVIS average radiance and field ratings for a bacterial spot in tomato genotypes inoculated with *X. hortorum* pv. *gardneri*.

**Tomato species**	**Genotype**	**IVIS^a^**	**Field^b^**
*S. lycopersicum* var. *cerasiforme*	PI 114490	−418.07	−1.31
*S. lycopersicum*	QTL-11A (+) *Xv3*	−372.39	−0.97
*S. lycopersicum*	QTL-11A only	−127.39	0.80
*S. lycopersicum*	QTL-11B (parent)	−69.92	−0.25
*S. pimpinellifolium*	LA2533	3.11	−1.38
*S. lycopersicum*	QTL-11A (parent)	19.27	−1.60
*S. lycopersicum*	QTL-11C (parent)	29.22	−2.05
*S. lycopersicum*	QTL-11C	52.93	−0.36
*S. lycopersicum*	QTL-11B	101.49	0.59
*S. lycopersicum*	*Xv3* only	200.72	1.21
*S. lycopersicum*	OH88119	581.03	2.62
	Linear correlation	*R*^2^ = 0.45	*p* = 0.024

a *IVIS = Best Linear Unbiased Predictors (BLUPs) of IVIS measured by average radiance (p/sec/cm^2^/sr). IVIS column represents data from four combined experiments using spray inoculated plants and adjusted to a mean of 0. Negative values reflect lower average radiance and therefore lower bacterial populations*.

b *Field = BLUPs based on bacterial spot field ratings of plants inoculated by spray, with values estimated from 4 years and adjusted to a mean of 0. Lower values reflect fewer symptoms, and therefore more resistant germplasm*.

Heritability and reliability are functions of the variance components and were greater for field ratings than for average radiance measured by IVIS. The statistical model using average radiance values showed that 25% of the total variance was explained by genetics. However, 63% of the total variance was explained by residual error. Replication within the experiment and between experiments explained very little variance in average radiance as measured by IVIS. The model using bacterial spot field rating data as the dependent variable showed that genotype differences (genetics) explained most of the variance, accounting for 63% of the total. The replication based on year explained 6% of the total variance, and the within-field variation effect was negligible. The residual error explained 30% of the variance for field rating data (Table 2). For average radiance data obtained from IVIS, heritability was 0.58 and reliability was 0.25. For field ratings, heritability was 0.86 and reliability was 0.63 (Table 2). Despite lower heritability and reliability, quantification of luminescent signals through IVIS may prove useful for conducting germplasm screenings during the off-season, for rapid confirmation of disease resistance, or experiments requiring rapid generation turnover. On average, IVIS evaluation of seedlings required 24 days from initial sowing to data acquisition. In contrast, field studies required 90–120 days.

**Table 2:** Variance partitioning of IVIS and field ratings using random models.

	**IVIS^a^**			**Field^b^**		
**Effect**	**Variance**	**Std. Dev**	**(%)Var**.	**Variance**	**Std.Dev**	**(%)Var**.
Genotype	106340.1	326.10	25%	2.28	1.51	63%
Experiment or Year	9663.3	310.91	2%	0.22	0.48	6%
Rep (Experiment or Year)	457.5	21.39	<0.001%	0.00	0.01	<0.0001%
Residuals	313806.0	560.18	73%	1.07	1.03	30%
Heritability^c^	0.58			0.86		
Reliability^d^	0.25			0.63		

a *IVIS, Phenotyping using (p/sec/cm^2^/sr) from IVIS, combined data from four experiments using spray inoculation. The proportion of variance was calculated for each term in the model*.

b *Field, Phenotyping using the Horsfall-Barrat Scale (1–12), 1 being most resistant. Combined data from 3 years of X. hortorum pv. gardneri field disease ratings with plants inoculated by spray. The proportion of variance was calculated for each term in the model*.

c *Heritability, Heritability was calculated as a function of variance components for each method of phenotyping using the formula $H=\;\frac{\sigma_{G}^{2}}{\sigma_{G}^{2}+\;\frac{\sigma_{E}^{2}}{n}}$ where $\sigma_{G}^{2}$ and $\sigma_{E}^{2}$ are the estimates of variance for the genotype and residual error and n is the number of experiments or years. The main effects for experiment and year are dropped*.

d *Realiabilty, Reliability was calculated as a function of variance components for each method of phenotyping according to the formula $i^{2}=\;\frac{\sigma_{G}^{2}}{\sigma_{G}^{2}+\sigma_{Y}^{2}+\sigma_{E}^{2}}$, where $\sigma_{Y}^{2}$ is the variance for either year or experiment*.

### The Tomato Leaf and Bacterial Colonization

**Figure 4:**
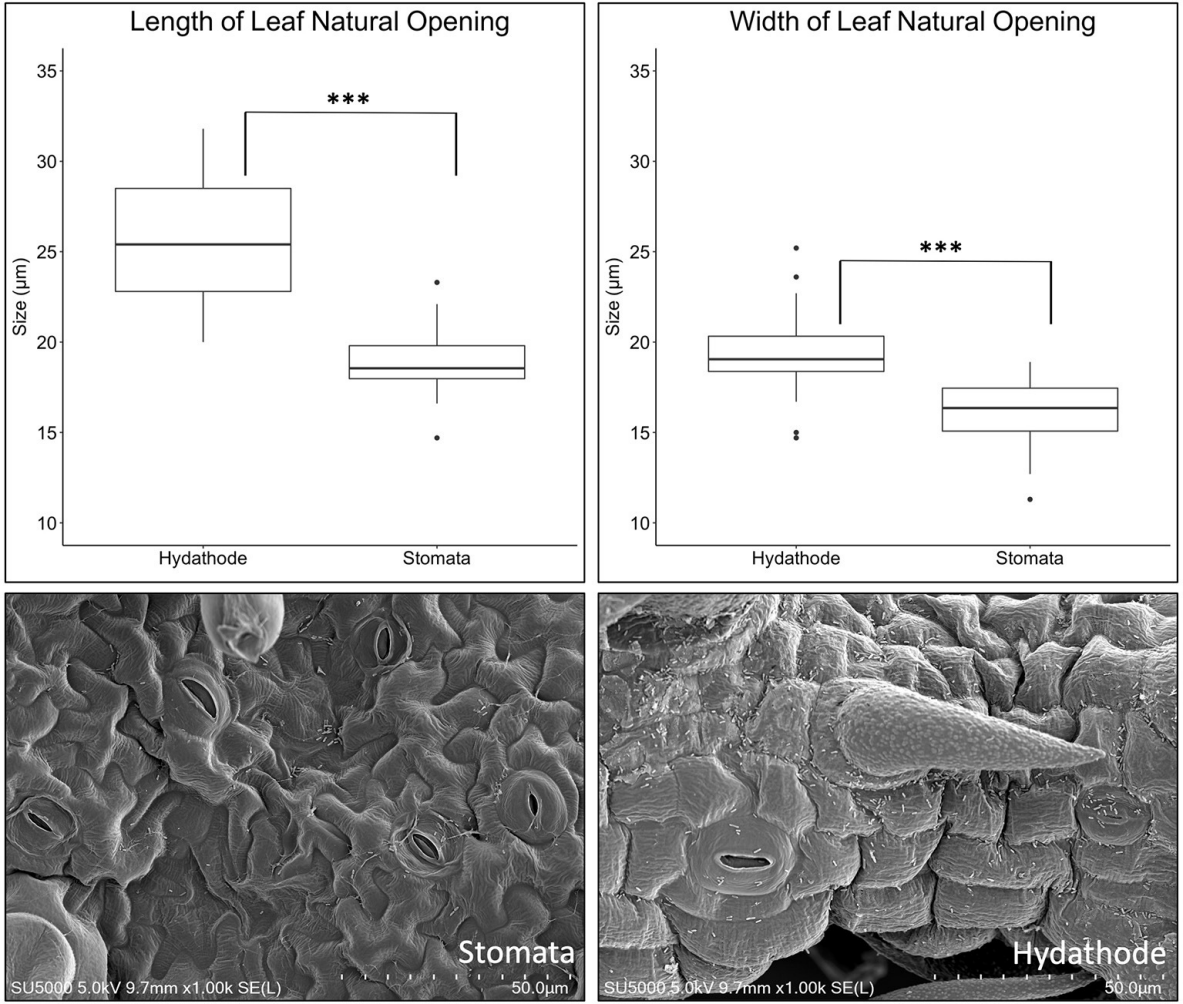
Differences in size between abaxial stomata vs. leaf margin hydathode pores of tomato line OH88119. The boxplots illustrate differences in size for abaxial stomata vs. leaf margin hydathode pores. Hydathode pores are greater in length and width compared to stomata (*P* < 0.0001***). Hydathode pores (right image) tend to have a more pronounced pore rim compared to stomata. Scanning electron microscopy (SEM) display *X. hortorum* pv. *gardneri* colonizing natural pores.

*In vivo* imaging system observations of average radiance due to *X. hortorum* pv. *gardneri* strongly implicate infection on the leaf margins and suggested hydathodes could be a point of entry. SEM provided an alternative method to observe and quantify bacterial location relative to natural openings on the leaf. The appearance of stomatal pores was similar, though significant differences in the size of stomata on the leaf surface and hydathodes on the leaf margin were observed (Figure 4). Hydathode pores had greater length and width than stomates, *F*_(1, 19)_ = 52.5, *p* = ≤ 0.001 and *F*_(1, 19)_ = 19.9, *p* ≤ 0.001. The average length (μM) and width (μM) of tomato hydathode pores were 25.66 ± 3.76 and 19.45 ± 2.61, respectively. The average length and width of tomato stomates were 18.86 ± 1.86 and 16.02 ± 1.96, respectively. In addition, hydathodes possessed a characteristic rise or rim (Figure 4) not observed on stomatal pores on the leaf surface.

**Figure 5:**
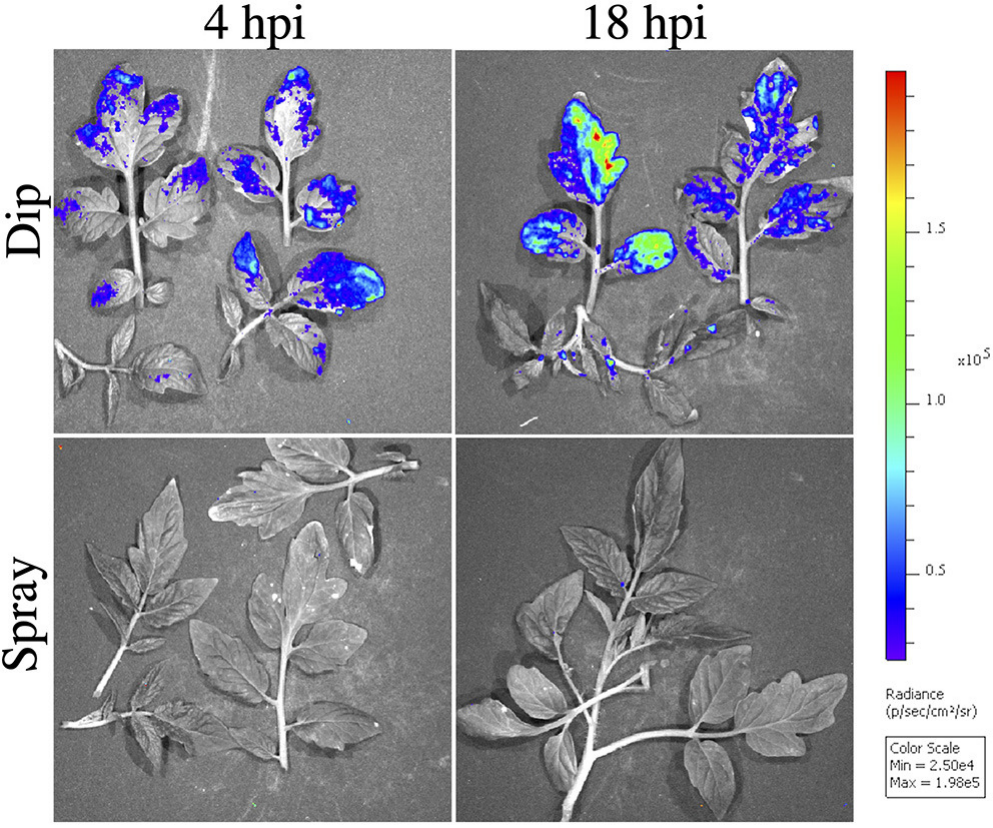
Observed *X. hortorum* pv. *gardneri* (Xg^b^) bacterial populations on tomato OH88119 leaf surfaces using *in vivo* imaging. OH88119 tomato seedlings were sprayed or dip inoculated with a suspension of Xg^b^, expressing the *lux operon*. Leaf surfaces were imaged *via* IVIS 4 h post-inoculation (hpi) and 18 hpi. No bioluminescence signal was observed at 4 or 18 hpi using spray inoculation, however, a bioluminescence signal was observed using dip inoculation. Strong bioluminescence signals were observed on leaf margins.

Bioluminescence detects the presence of Xg^b^ prior to symptom development. We observed no clear symptoms using spray inoculation through 9 days of observation. With dip inoculation using a surfactant, susceptible leaves often appear necrotic at 48–72 h. No bioluminescence signals were observed at 4 and 18 hpi with spray inoculation of Xg^b^. Signal detection often took 3 days when inoculated by spray. However, with dip inoculation containing surfactant, bioluminescence signals were observed at 4 and 18 hpi (Figure 5). Further, bioluminescence was detected across the entire leaf surface with dip inoculation (Figure 5), an observation that contrasted with the observations of infection patterns with spray inoculation where bioluminescence was first observed concentrated at the leaf margins at 72 h. The different distributions of bacteria based on the inoculation method observed using IVIS were also detected when quantifying bacteria from SEM images. A greater density of bacteria on leaf surfaces was observed when using dip inoculation vs. spray inoculation at 4 hpi, χ2 (1, *N* = 301) = 48.64, *p* < 0.0001. The same result was also observed at 18 hpi χ2 (1, *N* = 213) = 11.11, *p* < 0.0001. No significant differences were observed in the density of bacteria when comparing 4 and 18 hpi for dip inoculation, *F*_(1, 2)_ = 7.27, *p* = ≤ 0.11. When comparing the density of bacteria using the spray inoculation at 4 and 18 hpi we also did not see any significant differences, *F*_(1, 2)_ = 0.019, *p* = ≤ 0.70.

We compared the distribution of Xg^b^ on leaf surfaces at 4 hpi using dip or spray inoculation. At 4 hpi with dip inoculation containing surfactant, the distribution of bacteria was random on the leaf surfaces, χ2 (2, *N* = 209) = 5.29, *p* > 0.05. At 4 hpi with spray inoculation the bacteria were not randomly distributed, χ2 (2, *N* = 90) = 7.20, *p* < 0.05, with flat surfaces containing more bacteria per unit area (Table 3). At 18 hpi for spray inoculation, the distribution of bacteria was also not random and there was a shift toward a higher density observed on the leaf margins, χ2 (2, *N* = 75) = 26.96, *p* < 0.05. At 18 hpi with dip inoculation, we also observed a higher density of bacteria on the leaf margins, χ2 (1, *N* = 138) = 47.13, *p* < 0.05 (Figure 4). Dip inoculation in the presence of Silwett L77 resulted in a more rapid infection across the entire leaf surface by 4 h. With spray inoculation, bacteria concentrated on the leaf edges by 18 h, consistent with luminescence patterns, which indicated infection on the leaf margins by 3 days after inoculation.

**Table 3:** Chi-square test for *X. hortorum* pv. *gardneri* (Xg^b^) distribution on adaxial surface, abaxial surface, and leaf margin of OH88119.

**Time^a^ (hpi)**	**Inoculation method^b^**	**Df^c^**	**N^d^**	***X^**2**^***	***p*-value**	**notes**
4	Dip+Silwet	2	209	5.29	0.07	ns
	Spray	2	90	7.20	0.03	Excess on adaxial and abaxial surfaces
18	Dip+Silwet	2	138	47.13	<0.0001	Excess on leaf margin
	Spray	2	75	26.96	<0.0001	Excess on leaf margin

a *Time, leaf tissue was fixed for SEM and bacterial counts at 4 and 18 h post-inoculation (hpi)*.

b *Inoculation method = tomato plants were inoculated using either a dip in the presence of Silwet-77 surfactant or spray inoculation methods*.

c *df, degree of freedom for the chi-square test*.

d *N, total counts*.

## Discussion

Observations facilitated through bioluminescence have helped improve the understanding of biological interactions. Previous studies in various plant-pathogen systems have used bioluminescence to describe the infection processes (Dane and Dane, 1994; Azegami et al., 2004; Xu et al., 2010; Vrisman et al., 2016; Du et al., 2017). In this study, we utilized an *X. hortorum* pv. *gardneri* strain expressing the *lux operon*, Xg^b^, to study infection and assess disease severity in resistant and susceptible tomato lines. We showed increases in bacterial populations with a plateau between 5 and 7 days post-inoculation and significant correlations between bacterial counts measured by dilution plating and bioluminescence signals, demonstrating the ability to rapidly estimate bacterial populations *in planta*. There was variation experiment to experiment suggesting that although accurate relative estimates are possible, precise estimates of bacterial counts will require the generation of a standard curve for each experiment. A positive and significant correlation between bioluminescence signals and bacterial populations *in planta* has been observed in other plant-host interactions including *R. solanacearum* on pepper and potato, *C. michiganesis* subsp. *michiganesis* on tomato, and *X. campestris* pv. *vesicatoria* (now *X. euvesicatoria*) on tomato (Dane and Dane, 1994; Xu et al., 2010; Cruz et al., 2014; Du et al., 2017). This study describes *X. hortorum* pv. *gardneri* strain Xg^b^ as a resource.

Bioluminescent strains of various plant pathogens have been utilized to assess *in planta* growth dynamics on resistant and susceptible germplasm. For example, a bioluminescent *P. syringae* strain was used to monitor bacterial growth in resistant and susceptible bean lines (Paynter et al., 2006). Bioluminescence identified a dynamic in which bioluminescence signals decreased after 48 h in the resistant line but increased in the susceptible line. These results are interpreted as resistance suppressing bacterial populations early during infection. Bioluminescent *R. solanacearum* strains have been used to assess disease severity in potato germplasm and pepper lines (Cruz et al., 2014; Du et al., 2017). Bioluminescence patterns indicated the presence of bacteria in symptomless potato germplasm (Cruz et al., 2014). The marked strains permitted the detection of latent infection *via* bioluminescence 5 days post-inoculation. Infection of pepper by a bioluminescent *R. solanacearum* showed that resistant lines restricted bacterial multiplication in roots and stems (Du et al., 2017). Thus, the use of bioluminescence has provided insight into the time and location of infection.

We further explored the use of the bioluminescent *X. hortorum* pv. *gardneri* for rapid screening of germplasm. The use of IVIS to quantify bioluminescence signals allowed us to separate known resistant and susceptible lines. Screening based on total flux resulted in lower estimates of heritability and reliability relative to field screens, suggesting that the technique provided less accuracy in measuring genetic signals and for separating germplasm relative to classical field-based phenotyping. Plant breeders assess selection strategies gain under selection, gain per cycle, gain per unit time, and gain per dollar invested in screening. The practicality of IVIS as a screening method, therefore, needs to consider the relative efficiency of selection. Relative efficiency per cycle of selection is 0.53, calculated as $RE=r_{g}^{*}\frac{h_{I}}{h_{F}}$ where r_g_ is the genetic correlation between the BLUPs estimated from total flux measured by IVIS and *X. hortorum* pv. *gardneri* field rating, h_I_, and h_F_ are the square root heritabilities of IVIS and field rating BLUPs. Selection using IVIS is therefore expected to be 53% as efficient as a direct selection in the field per cycle of selection. Because screening germplasm *via* IVIS only requires 30–40 days compared to 90–120 days in the field, it is possible to increase cycles of selection on a per-year basis. The tomato reproductive cycle would be the only limitation to screening rather than available field seasons in any given year. IVIS is a tool proved to be efficient in terms of time for phenotyping disease resistance and the method may have a role in future germplasm screens for this reason alone.

It may be possible to increase the heritability of disease screening using IVIS by accounting for morphological features and growth. Tomato plants grow at different rates and possess distinct leaf morphologies, especially wild tomato germplasm. Digital methodologies that account for leaf area could be incorporated in future analyses. In these studies, we initially used NILs, which are highly genetically similar and therefore differences in growth rate or leaf morphology were not an issue. However, when we expanded the selection of germplasm to include wild species, error variance increased.

In germplasm screening experiments using spray inoculation, we observed strong bioluminescence signals at the leaf margins suggesting that *X. hortorum* pv. *gardneri* colonized hydathodes. We used microscopic observations to obtain quantitative data of *X. hortorum* pv. *gardneri* distribution on leaf surfaces. Spatial statistics in combination with microscopy has been a useful tool to study patterns of microbial colonization on the plant rhizosphere and phyllosphere (Dandurand et al., 1997; Remus-Emsermann et al., 2014; Schmidt et al., 2018). The statistical analyses use a formal hypothesis to test for random patterns on a specific location or surface (Schmidt et al., 2018). We applied similar approaches to quantify bacterial distributions on leaf surfaces infected with *X. hortorum* pv. *gardneri*. We observed differences in bacterial distribution on leaf surfaces at 4 hpi using different inoculation methods (spray vs. dip with surfactant). At 4 hpi using dip inoculation in the presence of surfactant, bacteria were randomly distributed on leaf surfaces, but the distribution was non-random following spray inoculation. This observation could suggest that bacteria are adhering to all leaf surfaces when dipped, but when sprayed the bacteria showed greater density on the abaxial and adaxial sides compared to the leaf edge. However, at 18 hpi both spray and dip inoculation showed a higher density of bacteria on leaf edges. The differences were observed between bacterial distributions due to inoculation methods, the more rapid detection of bacterial luminescence with the surfactant, and more rapid detection of symptoms with surfactant suggest that inoculation methods need to be considered when interpreting results. The IVIS observations were supported by SEM analysis showing that 18 h post spray inoculation, the distribution of bacteria would support ingress through hydathode water pores. These water pores are longer and wider than stomata and tend to exude extracellular fluid containing sugars, vitamins, and other solutes that may promote a conducive environment for bacterial growth and colonization (Cerutti et al., 2019). The distribution of bacteria as quantified by SEM and bioluminescence patterns suggest hydathode pores may be entry points for *X. hortorum* pv. *gardneri*.

## Data Availability Statement

The raw data supporting the conclusions of this article will be made available by the authors, without undue reservation.

## Author Contributions

DF established the experimental design and assisted with data analyses. EB established the experimental design, performed all inoculations, collected, and analyzed data. LD and GR developed the bioluminescent strain and provided support for the use of *in vivo* imaging system. All authors contributed to the article and approved the submitted version.

## Conflict of Interest

The authors declare that the research was conducted in the absence of any commercial or financial relationships that could be construed as a potential conflict of interest.
